# Poor self-rated health did not increase risk of permanent nursing placement or mortality in people with mild Alzheimer’s disease

**DOI:** 10.1186/s12877-016-0262-x

**Published:** 2016-04-19

**Authors:** Anni Brit Sternhagen Nielsen, Volkert Siersma, Gunhild Waldemar, Frans Boch Waldorff

**Affiliations:** The Research Unit and Section of General Practice, Institute of Public Health, University of Copenhagen, Copenhagen, Denmark; Danish Dementia Research Centre, Department of Neurology, Rigshospitalet, University of Copenhagen, Copenhagen, Denmark; The Research Unit for General Practice, Department of Public Health, University of Southern Denmark, Copenhagen, Denmark

**Keywords:** Alzheimer Disease, Cohort studies, Mortality, Nursing homes, Self-rated health

## Abstract

**Background:**

Self-rated health (SRH) has in many population-based studies predicted adverse health outcomes, e.g. morbidity, permanent nursing home (NH) placement, and mortality. However, the predictive value of SRH to NH placement and mortality among elderly people is not consistent. This may be due to cognitive impairment. Since the SRH item is widely used, it is important to know whether SRH has different predictive value among people with cognitive impairments. We aimed to examine SRH and the risk of permanent NH placement and mortality among people with mild Alzheimer’s disease (AD).

**Methods:**

Data are from The Danish Alzheimer Intervention StudY (DAISY), a large randomized controlled trial of psychosocial intervention for patients with mild dementia and their caregivers with 3-years’ follow-up. Five out of 14 Danish counties participated and 321 home-living elderly (mean age: 76.2 years) with mild AD (46.4 % male) were included during 2004 and 2005. Self-rated SRH, cognitive function (MMSE), quality of life (proxy-rated QOL-AD), activities of daily living (ADCS-ADL), insight, and socio-demographics were assessed at baseline. Comorbidities and information about NH placement and mortality was obtained over 3-years’ follow-up from registries. With Cox proportional hazard regression we analysed the association between SRH (dichotomised into good vs. poor) and NH placement and mortality adjusted for potential confounders.

**Results:**

At baseline 66 % reported excellent or good, and 34 % fair, poor or very poor SRH. Mean MMSE was 24.0 (range: 20–30). NH placement and mortality totalled 28.1 % and 16.5 % at 3-years’ follow-up, respectively. Poor SRH at baseline was not related to increased risk of NH placement or to increased mortality neither in the univariable nor in multivariable analysis: In the fully adjusted models HR was 0.63 (95 % CI 0.38-1.05) and 1.28 (95 % CI 0.67-2.45), respectively.

**Conclusions:**

When poor SRH was present we found no increased risk for NH placement or death among elderly people with mild AD. SRH is a widely used parameter in clinical and epidemiological research but may not be a valid indicator of health in patients with AD due to loss of insight.

## Background

Self-rated health (SRH) measured by a single question has in many population-based studies shown to be an independent predictor of future adverse health outcomes such as nursing home (NH) placement [1] and mortality [2–4] even when accounting for possibly health factors like lifestyle, socio-economic status, and co-morbidities.

However, the predictive value of SRH to both NH placement and mortality among elderly people is unclear: some studies have shown that poor SRH as compared to good SRH predicted future NH placement [1, 5–8] also if information on cognition was included [1, 7, 8], while other studies also including information on cognition, and thereby had included cognitive impaired persons in the studies, found no relation between SRH and NH at all [9–13].

Several reviews of community-based cohort studies have found that higher risk of mortality was associated with worse SRH [2, 3]. However, four community-based cohort studies including elderly aged 65 years or older also adjusted the analysis for information on cognitive function [14]; re-analysis of those four studies in a meta-analysis did not show the expected relation between SRH and mortality: a higher relative risk of mortality was found among people who rated their health good as compared to excellent, but no higher risk was found among those who rated their health fair or poor [14]. Conversely, another study including of Canadian community-dwelling elderly (>65 years) found that SRH was a valid predictor also among those with mild to moderate cognitive decline, but not among those with severe cognitive impairment [15]. An article discussing the future research into a better understanding of SRH also calls for further studies on cognitive processes of health assessments [4].

Since the SRH item is widely used, it is important to know whether SRH has different predictive value for NH placement and mortality in people with cognitive impairments. We hypothesize that those with poor SRH at baseline have an increased risk of NH placement and mortality during 3-years of follow-up.Therefore the present study aimed to examine SRH and the risk of permanent NH placement and mortality during 3-years of follow-up among people with mild Alzheimer’s disease (AD).

## Methods

### Study population

This post hoc study used data from the Danish Alzheimer Intervention StudY (DAISY) [16–18], a longitudinal rater-blinded randomized multicenter study, which compared usual care to the efficacy of an intensively structured intervention program with education, counselling and support to home-living people with mild AD, and their primary caregiver. The participants were enrolled from April 2004 until August 2005 through mailed announcements to relevant clinics and physicians among five Danish regions: one urban, three rural, and one mixed.

Inclusion criteria were ≥ 50 years old, home-living, clinical diagnosis (<12 months) of probable AD, mixed AD [19, 20], or dementia with Lewy bodies (DLB) [21] established or confirmed by the local specialist referral unit (memory clinic), and an mini mental state examination (MMSE) ≥ 20 [22]. Furthermore, participants should have at least weekly contact with a caregiver willing to participate. Exclusion criteria were participation in other intervention studies either at inclusion time or during the study, severe somatic or psychiatric co-morbidity as well as impaired vision or hearing which would considerably hamper cooperation with the program.

### Outcome

The end-points were permanent NH placement and all-cause mortality within 3 years from project inclusion.

### Measurements

Comprehensive information on the methods used to examine the participants at baseline have previously been reported [16]. In brief, the following measurements were done:

1. A structured interview of the participant performed by the local project-coordinator concerning aspects of health, social relation, daily life, driving, and legislation. The SRH-item was from the SF-36 health questionnaire “In general, would you say your health is” and had five response categories: excellent, good, fair, poor and very poor [23]. Participants with health ratings good or excellent were defined as having ”good” SRH, and those with fair/poor/very poor were defined as having “poor” SRH. Information on social participation within the last months was provided by the caregiver and measured by three questions “how often did he/she” (a) have visitors at home? (b) visit others? and (c) participate in social activities outside home? This three-item scale describes the participant’s social participation [24].
2. The Mini Mental Status Examination (MMSE) was used to assess global cognitive function, range 0–30 points. Higher scores indicate better cognitive function [22]. The Alzheimer’s Disease Co-operative Study – Activities of Daily Living Inventory (ADCS-ADL), was used to assess functional ability, based on caregiver interview (range 0–78). Higher scores indicate better functioning [25].
3. The Quality of Life-Alzheimer's Disease (QOL-AD), a 13-item questionnaire, was used to assess proxy-rated quality of life [26]. It was completed by the primary caregiver in order to get a measurement not affected by loss of insight. The scores range between 13–52; higher scores indicate better QOL.
4. The local project-coordinator completed the Anosognosia Rating Scale based on the interviews and cognitive testing with the participant as well as the caregiver. The participant’s level of insight was rated on a categorical four point scale [27]. Insight was classified into: “full”, “shallow”, “none”, and “denies impairment”. Furthermore, the project-coordinator interviewed the patient and the caregiver and completed the Cornell scale for Depression in Dementia (depression) [28] range 0–38. Higher scores indicate more depressive symptoms. Scores <6 indicate no depressive symptoms; scores = > 6 indicate depression, and scores > 10 indicate possible major depression [28].

### Registry data

Information on permanent NH placement status and incident deaths was given by the local project-coordinators, the caregivers, and the National Health Register. A Charlson’s Comorbidity Index (CCI) based on register data of hospital visits from the National Patient Registry in the time period up to three years before the inclusion date was made. CCI measures the level of comorbidity with regard to its impact on mortality [29].

### Ethics and consent

According to the Danish Act on Research Ethics, approval from the regional ethical committee was not required. But, the protocol was presented to the regional ethical committee for Copenhagen and Frederiksberg municipalities (The Scientific-Ethical Committee for Copenhagen and Frederiksberg Municipalities, Koebenhavns Sundhedsdirektorat, Postboks 620, Sjaellandsgade 40, DK-2200 Copenhagen). The committee reported that no approval was needed (ID No (KF) 02-005/04). The Danish Data Protection Agency approved the DAISY project (ID No 2003-41-3178) and the project was registered in the Clinical Trial Database (ISRCTN74848736). All participants and caregivers gave informed consent to study participation.

### Statistical analyses

We analysed differences in baseline characteristics and NH placement and death over 3-years’ follow-up between participants who rated their health good vs. poor with χ^2^- tests.

We examined the unadjusted association between SRH and other risk factors measured at baseline, i.e. socio-demographic characteristics, the number of comorbidities, MMSE, and other disclaims as well as randomisation group and county, and NH placement and death over 3-years’ follow-up with Cox proportional hazard models. The effect was assessed as a Hazard Ratio (HR) with a 95 % confidence interval (CI); corresponding to a certain category of the risk factor, i.e. the event rate in that category compared to the event rate in a baseline category.

In three multivariable Cox proportional hazard models, we investigated whether an effect of SRH was present on permanent NH placement and death, respectively, over 3-years’ follow-up adjusted for possibly confounding factors. Model I was only adjusted for diagnosis, comorbidities, social participation, sex, age, marital status, education, randomisation group and county. Model II included the variables in Model 1 and adjusted additionally for depression, proxy-rated QOL-AD and ADCS-ADL. Finally, Model III included the variables from Model II together with information on MMSE and insight.

Overall comparison of the three multivariable models for either permanent NH placement or death, was done by examination of the information criteria AIC and SBC; the lowest score indicating the best model.

All analyses were performed in SAS, version 9.1 (SAS Institute Inc, Cary, NC). The nominal statistical significance level was <0.05.

## Results

A total of 321 (45.2 % men) participants with mild Alzheimer’s disease were included. At baseline 212 (66 %) had excellent or good SRH, and 109 (34 %) had fair, poor or very poor SRH. The average age and MMSE of participants was 76.2 years (range 54–92) and 24.0 (range: 20–30), respectively. Table 1 shows demographic and clinical characteristics stratified by SRH. At 3-years’ follow-up permanent NH placement totalled 89 (21.1 %) and deaths 52 (16.5 %). Women and participants with low educational level, depression, full or shallow insight versus none were more prone to rate their health as poor; and participants with a MMSE score less than 27 were more prone to rate their health good as compared to those with a higher MMSE score.

**Table 1:** Baseline characteristics, NH placement and death of participants in the Danish Alzheimer Intervention StudY (DAISY)

		Self-rated health							
		good (n = 212)		poor (n = 109)					
		n	%	N	%	χ^2^	DF	*P*-value	Missing
Nursing home placement^a^	No	147	69.7	81	76.4	1.59	1	0.21	4
	Yes	64	30.3	25	23.6				
Death^a^	No	180	84.9	88	80.7	0.91	1	0.34	0
	Yes	32	15.1	22	19.3				
Sex	Women	107	50.5	69	63.3	4.79	1	0.03	0
	Men	105	49.5	40	36.7				
Age	50-74 years	82	38.7	38	34.9	0.45	1	0.50	0
	≥75 years	130	61.3	71	65.1				
Diagnosis	AD	160	75.5	79	72.5	0.34	1	0.56	0
	Mixed AD/VaD^b^	52	24.5	30	27.5				
Living alone	No	149	70.3	74	67.9	0.19	1	0.66	0
	Yes	63	29.7	35	32.1				
Education	≥3 years	99	46.7	35	32.1	6.30	1	0.01	0
	<3 years	113	53.3	74	67.9				
Social participation	High	103	51.8	58	57.4	0.87	1	0.35	21
	Low	96	48.2	43	42.6				
Comorbidity scale^c^	0	86	40.6	51	46.8	2.42	2	0.30	0
	1	93	43.9	38	34.9				
	≥2	33	15.6	20	18.4				
Depression scale^d^	Not depressed	155	73.1	62	56.9	8.66	1	0.003	0
	Depressed	57	26.9	47	43.1				
QOL-AD	30-52 (high)	164	77.4	80	73.4	0.62	1	0.43	2
	13-29	48	22.6	29	26.6				
ADCS-ADL	60-78 (high)	130	61.3	71	65.1	0.45	1	0.50	0
	0-59	82	38.7	38	34.9				
MMSE	27-30 (high)	36	17.0	25	22.9	2.24	2	0.33	0
	24-26	79	37.3	42	38.5				
	20-23	97	45.8	42	38.5				
Insight (anosognosia)	Full	59	27.3	37	34.3	13.93		0.0009	1
	Shallow	120	56.6	69	63.9				
	None	33	15.6	2	1.8				
Randomization group	Control	106	50.0	59	54.1	0.49	1	0.48	0
	Intervention	106	50.0	50	45.9				

^a^Incidence within 36-monts’ follow-up

^b^Mixed AD and vascular dementia

^c^Charlson’s comorbidity index calculated on hospitalization diagnosis up to three months before baseline

^d^The Cornell scale for Depression in Dementia, Depressed = (Cornell > =6)

In the unadjusted analyses we saw no higher prevalence of NH placement and death among those with poor SRH as compared to good SRH (Tables 1 and 2): HR was 0.74 (95 % CI 0.47-1.28) and 1.30 (95 % CI 0.75-2.25), respectively. Table 2 shows the effects between SRH and each risk factor individually on NH placement and death, and as can be seen no other included factors such as age, sex, and comorbidities gave an increased risk.

**Table 2:** Unadjusted relation between SRH and other risk factors on NH placement and death over 3-years’ follow-up

		Nursing home placement					Death				
Adjusted for	Self-rated health	HR	95 % CI		χ^2^	*P*-value	HR	95 % CI		χ^2^	*P*-value
Sex	Good	1.00			2.93	0.09	1.00			1.12	0.29
	Poor	0.67	0.42	1.06			1.35	0.77	2.36		
Age	Good	1.00			2.07	0.15	1.00			0.76	0.38
	Poor	0.71	0.45	1.13			1.28	0.74	2.22		
Diagnosis	Good	1.00			1.78	0.18	1.00			0.83	0.36
	Poor	0.73	0.46	1.16			1.29	0. 75	2.24		
Living alone	Good	1.00			1.86	0.17	1.00			0.81	0.37
	Poor	0.73	0.46	1.15			1.29	0. 74	2.24		
Education	Good	1.00			2.44	0.12	1.00			1.12	0.29
	Poor	0.69	0.43	1.10			1.35	0.77	2.36		
Social participation	Good	1.00			1.43	0.23	1.00			0.06	0.81
	Poor	0.75	0.47	1.20			1.07	0.59	1.94		
Comorbidity scale	Good	1.00			1.56	0.21	1.00			0.89	0.35
	Poor	0.74	0.47	1.18			1.31	0.75	2.27		
Depression scale	Good	1.00			2.50	0.11	1.00			0.79	0.37
	Poor	0.68	0.43	1.10			1.29	0.74	2.26		
QOL-AD	Good	1.00			1.91	0.17	1.00			0.80	0.37
	Poor	0.72	0.45	1.15			1.29	0.74	2.23		
ADCS-ADL	Good	1.00			1.41	0.24	1.00			1.42	0.23
	Poor	0.76	0.48	1.20			1.40	0.81	2.43		
MMSE	Good	1.00			1.07	0.30	1.00			0.94	0.33
	Poor	0.78	0.49	1.24			1.31	0.76	2.28		
Insight (anosognosia)	Good	1.00			1.43	0.23	1.00			1.40	0.24
	Poor	0.75	0.47	1.20			1.46	0.80	2.51		
Randomization group	Good	1.00			1.66	0.20	1.00			0.98	0.32
	Poor	0.74	0.47	1.17			1.32	0.76	2.29		
County	Good	1.00			1.63	0.20	1.00			0.46	0.50
	Poor	0.74	0.46	1.18			1.21	0.70	2.11		

Table 3 shows three different multivariable Cox regression models for NH placement and death, respectively. In all the models we found no increased risk for NH placement or death among those rating their health poor as compared to good; in the fully adjusted models HR was 0.63 (95 % CI 0.38-1.05) and 1.28 (95 % CI 0.67-2.45), respectively. Model III gave the best explanatory power for NH placement while model II was best for mortality. For NH placement we found that very low scores of MMSE (HR = 2.40 for MMSE 20–23 vs reference 27–30) gave an increased risk, but not insight. For mortality, in Model II, we also found that insight did not increase the risk.

**Table 3:** Multivariable assessment of the effect of SRH on NH placement and death over 3-years’ follow-up

		Model I^a^					Model II^b^					Model III^c^				
		Hazard ratio	95 % CI		χ^2^	*P*-value	Hazard ratio	95 % CI		χ^2^	*P*-value	Hazard ratio	95 % CI		χ^2^	*P*-value
Permanent NH placement																
SRH	good	1			3.68	0.06	1			4.06	0.04	1			3.18	0.07
	poor	0.61	0.37	1.01			0.60	0.36	0.99			0.63	0.38	1.05		
AIC^d^	912.175						901.278					898.965†				
SBC^d^	956.207						942.602					950.012†				
Death																
SRH	good	1			0.05	0.82	1			0.32	0.57	1			0.58	0.45
	poor	1.08	0.58	1.99			1.20	0.64	2.23			1.28	0.67	2.45		
AIC^d^	549.708						533.353†					539.046				
SBC^d^	575.905						565.164†					578.341				

^a^Adjusted for diagnosis, comorbidities, social participation, sex, age, marital status, education, randomisation group, and county

^b^ Adjusted for the variables included in Model 1 and additionally for depression, QOL-AD and ADCS-ADL

^c^Adjusted for the variables included in model II and additionally for MMSE and insight

^d^AIC and SBC based on data without missing values for all risk factors

† Best model according to AIC and SBC over model I, II and III

## Discussion

We found that poor SRH was not associated with an increased risk of NH placement or mortality during 3- years’ follow-up among home-living participants suffering from mild AD.

By using the administrative registers, and the information from caregivers and project-coordinators we were able to account for all death and almost all NH placements (information missing on 5 people) among the participants. SRH was measured with a single widely used item for prediction of adverse outcomes [30]. We did not have access to databases regarding medication, which would have been relevant to evaluate [31], however we included information on comorbidities (through the CCI index) which may possibly reflect use of medication. One of the study’s strengths was the well-defined population of participants suffering from mild AD based on internationally accepted criteria, using validated assessment scales developed for people with dementia. In addition we accounted for potential confounding factors in the multivariable analyses, e.g. educational level, age, Quality of life, ADL, comorbidities, depression, MMSE, and insight in own deficits.

In both the univariable and the multivariable analyses of the relation between SRH and NH placement we found that poor SRH did not increase the risk of NH placement (Model III), a finding that is in line with other findings among elderly people suffering from cognitive impairment [9–13]. A possible explanation for this result could be lack of insight (anosognosia). Based on result of the anosognosia scale we found, that the participants least aware of their cognitive impairment were more prone to rate their health good. It is understandable that insight is crucial when rating own health and function: results from qualitative interviews have shown that responder’s reason for their own health rating may represent a personal estimation of longevity [32]. Other factors attributed to an individual health rating are current or previous physical health, perception of symptoms, physical functioning and personal resources including present health behaviour [33, 34] knowledge of possible familial disposition [32], and comparison with health of age peers [34]. Those with low MMSE scores (less than 20–24) had a higher risk of NH placement than the reference group, even though they had a tendency of rating their health good. The latter may indicate that the cognitive skills people in general use when rating their health has declined [35]. The finding of a higher risk of NH placement among those with dementia is in accordance with several other studies [11, 36–38].

We found no relation between SRH and death, neither in the unadjusted nor in multivariably adjusted analyses. This finding is in accordance with a Canadian study examining survival among elderly (≥65 years): SRH did not predict survival among participants with severe cognitive impairment [15]. In our study we found that the participants tended to rate their health good when being more cognitively impaired, e.g. 59 % with a MMSE score >27, 65.3 % and 69.8 % of those with a MMSE score between 24–26 and 20–23, respectively, rated their health good. The same reverse tendency of rating health good when cognitive function declined was seen for insight in own deficit. An Australian [39] and a Danish [40] study of elderly above 70 and 80 years, respectively, also included information on cognitive functioning in their analyses of the association between SRH and mortality. They too found no relation at all. However, if they performed the analyses separately for men and women the Australian study found that SRH was a predictor of death among men while the opposite were seen in the Danish study (women). As opposed to the Australian and Danish study we found no higher mortality risk among men as compared to women in the unadjusted analyses; and our multivariably adjusted analyses were not split by sex since this was not the purpose of the study The lack of association between SRH and mortality in our sample of participants with mild AD may possibly reflect that SRH is not a good measurement of the participants’ mental disease severity, which may indicate that the cognitive skills people in general use when rating their health has declined [35]. It seems that proxy-rated quality of health among the patients with mild dementia is a more useful predictor of mortality. In another study, including the same patients as in our study, a dose–response relation between the caregiver’s rating of the quality of health of the patient on the Euro-QoL visual analogue scale and the patient’s future mortality [41].

## Conclusion

In a 3-years’ follow-up study of elderly people diagnosed with mild AD who provided ratings of their health at baseline (SRH), we found no higher risk of permanent NH placement or death among those with poor SRH. We hypothesize that our result is due to lack of insight (anosognosia); those with lack of insight are more prone to rate their health good. We recommend future studies to be aware that SRH, included in many questionnaires, may be a good instrument in population studies and perhaps among other patient groups, but due to the reduced insight not a good indicator of vulnerability among people suffering from mild AD. Since proxy-rated health, as predictor of mortality in patients with mild dementia, may provide information on future death we recommend future studies to examine the validity of proxy-rated health of life.

### Availability of supporting data

Data files are not available due to participants’ confidentiality.

## Abbreviations

95 % CI: 95 % Confidence Interval
AD: Alzheimer’s disease
ADCS-ADL: The Alzheimer’s Disease Co-operative Study – Activities of Daily Living Inventory
CCI: Charlson’s Comorbidity Index
CI: Confidence Interval
CSDD: Cornell Scale for Depression in Dementia
DAISY: Danish Alzheimer Intervention StudY
DLB: Dementia with Lewy bodies
HR: Hazard Ratio
MMSE: Mini mental state examination
NH: Nursing home
QOL-AD: Quality of life scale for people with Alzheimer’s disease. In this paper a proxy-rated QOL-AD was used
SRH: Self-rated health
